# Unmuting Medical Students’ Education: Utilizing Telemedicine During the COVID-19 Pandemic and Beyond

**DOI:** 10.2196/19667

**Published:** 2020-07-20

**Authors:** Ariella Magen Iancu, Michael Thomas Kemp, Hasan Badre Alam

**Affiliations:** 1 University of Michigan Medical School Ann Arbor, MI United States; 2 Department of Surgery University of Michigan Ann Arbor, MI United States

**Keywords:** telemedicine, medical education, medical school, COVID-19, education, medical, undergraduate, curriculum

## Abstract

Due to the coronavirus disease (COVID-19) pandemic, medical schools have paused traditional clerkships, eliminating direct patient encounters from medical students’ education for the immediate future. Telemedicine offers opportunities in a variety of specialties that can augment student education during this time. The projected growth of telemedicine necessitates that students learn new skills to be effective providers. In this viewpoint, we delineate specific telehealth opportunities that teach core competencies for patient care, while also teaching telemedicine-specific skills. Schools can further augment student education through a variety of telemedicine initiatives across multiple medical fields. The explosion of telemedicine programs due to the pandemic can be a catalyst for schools to integrate telemedicine into their current curricula. The depth and variety of telemedicine opportunities allow schools to continue providing high-quality medical education while maintaining social distancing policies.

## Medical Education on Hold

On March 23, 2020, the Association of American Medical Colleges (AAMC) issued guidance on medical student involvement during the coronavirus disease (COVID-19) pandemic, recommending that medical students not participate in direct patient care, unless there is a critical workforce need, and only on a voluntary basis [1]. The COVID-19 pandemic has generated unprecedented stress for our national health care system. Consequently, it has created practical and logistical challenges for the learning environment that can have a lasting impact on medical education [2]. The widely varying predictions, regarding how long the pandemic will continue, raise questions as to when and how traditional clerkships will resume. In light of this, academic centers must consider new measures to continue their mission of training future physicians. There are potential technological solutions to the current challenge of clinical education during a time of social distancing.

The cornerstone of medical education is patient care. While direct patient care has been appropriately limited at most schools, the lessons learned from these critical patient interactions cannot be fully replaced by readings, lectures, case studies, or online modules. Certainly, direct in-person care has important educational value to the medical student. Although not an exact replacement for in-person care, institutions have a unique opportunity to bridge this current gap in clinical education with telemedicine. This viewpoint contributes to the ongoing discussion by offering specific guidance on how schools can incorporate medical students into the massive expansion of telehealth initiatives during and beyond the COVID-19 pandemic.

## The Expansion of Telemedicine

In recent years, telemedicine has grown exponentially. Telemedicine applications have expanded from phone triage and ambulatory electronic visits (e-visits) to include mental health, postsurgical operation follow-ups, and specialty consults [3]. In support of the efforts to socially distance medical care during the COVID-19 crisis, health systems across the United States are ramping up their telehealth programs [4]. As both patients and providers grow more accustomed to these virtual interfaces during the pandemic, many will likely choose to continue using telemedicine going forward [5]. Even before the pandemic, the telemedicine market was predicted to grow from $38.3 billion in 2018 to $130.5 billion by 2025 [3]. Therefore, while telemedicine has seen an acute spike in response to the pandemic, it is anticipated that it will continue to be an important vehicle for health care.

## A Unique Skillset for the Telemedicine Provider

With the imminent growth of telemedicine, teaching providers this unique skillset is essential for the success of future telemedicine programs. Practitioners who have not used telemedicine may find it difficult to translate their traditional interview and physical exam skills to the virtual sphere [6]. Furthermore, technical challenges are cited as one of the most common reasons for failure to implement telemedicine by providers [7]. These barriers are compounded by patient discomfort, possible technological illiteracy, and the stress associated with adapting to a new environment or system [6]. Additionally, a computer screen or phone makes for more detached patient interactions and distant provider-patient relationships [6]. When providers do not have the relevant skills, it becomes harder to concurrently adopt new technologies and maintain high-quality patient care. One study has found that trainees inexperienced with telemedicine struggled with gaining detailed histories or providing appropriate next steps—jumping straight to requesting unnecessary in-person visits [8]. A systematic review has found that courses have been successful in teaching telemedicine skills through dedicated curricula [9]. With the proper resources, these skills can be taught and therefore should become a key component of contemporary medical school curricula.

## Telemedicine Curriculum Considerations

Education is critical to the future success of telemedicine implementation. Integration of telemedicine into undergraduate medical education allows for all future physicians to have access to this type of training. The American Medical Association (AMA) has similarly articulated the value of telemedicine curricula in medical schools and residency programs [10]. In addition, the United States Medical Licensing Exam incorporates telephone encounters into its Step 2 Clinical Skills exam [11]. Multiple curricular resources already exist that have identified core competencies for telemedicine for physicians [6,12]. Other health professions have even initiated discussions around establishing competencies associated with virtual care [13].

There are a number of suggested skills associated with optimal telemedicine care. These include but are not limited to the following: communication, physical examination, professionalism, and technological literacy. Communication skills include clear enunciation and the minimization of body motions, gestures, and colloquial speech [12]. These measures help to account for clarity that may otherwise be lost through online platforms [6]. Unique physical exam techniques that can be learned involve functional physical exams [6], application of remote monitoring devices [12], and collaboration with on-site providers. Virtual evaluations can extend to include home assessments, such as in-home mobility barriers [6]. Training for professionalism in a virtual domain can cover education on privacy concerns, electronic prescribing (e-prescribing), and reporting of practices [6,12]. Trainees should also learn technological skills such as screen sharing to discuss diagnostic findings [6], assessing for technological literacy [6], and coaching patients through the use of virtual health platforms [12].

Some programs have already started to institute courses to teach these telemedicine communication and evaluation skills. Among the most common curricular elements includes standardized patients [8,9]. However, creating simulations, training standardized patients, and implementing new technology may prove to be insurmountable barriers for medical schools in the current setting. Instead of recreating new technology solely for medical students, schools can engage students in clinical care through the same telemedicine technology that hospital systems are currently employing. Already, a select number of schools successfully expose students to telemedicine during traditional clinical rotations [9]. With the acute expansion of telemedicine, more schools could provide enriching telemedicine opportunities for their students.

## Envisioning Remote Clinical Education During COVID-19

Telemedicine curricula could mimic traditional curricula that medical educators already employ with minimal adjustments, allowing for the continuation of clinical education during the pandemic. Students can easily access and get involved with telemedicine initiatives through technological platforms. For example, some electronic health record (EHR) platforms already integrate third-party platforms that would allow three-person calls [14]. Other options could be to implement Health Insurance Portability and Accountability Act (HIPAA)–compliant group-video options [15], or a three-way call led by the student. Third-party platforms often require institution licenses; however, with the dramatic growth in the last month, many of these platforms have become more readily available and have expanded their services [15]. Future access may require advocacy from medical schools for student licenses, or for clinician educators to share licenses with their students.

Educating students through telehealth initiatives could parallel a majority of the 13 Core Entrustable Professional Activities for entering residency (EPAs) (Table 1). These EPAs were created by the AAMC to provide medical schools with curricular guidelines to ensure a uniform set of skills for all medical school graduates [16]. We identified several curricular activities for schools to integrate now and during the transition back to regular clerkships.

**Table 1:** Telemedicine curricular activities that align with each of the Association of American Medical Colleges’ Core Entrustable Professional Activities (EPAs) [16].

EPA	Telemedicine curricular activity
EPA 1: Gather a history and perform a physical exam	Clinical e-visit^a^ Virtual consult
EPA 2: Prioritize a differential diagnosis	Clinical e-visit Virtual consult Pathology/radiology cases
EPA 3: Diagnostic and screening tests	Clinical e-visit Virtual consult Pathology/radiology cases
EPA 4: Enter and discuss orders and prescriptions	Clinical e-visit Virtual consult
EPA 5: Document a clinical encounter	Clinical e-visit Virtual consult Pathology/radiology cases
EPA 6: Provide an oral presentation of a clinical encounter	Clinical e-visit Virtual consult Pathology/radiology cases Student-led patient education project
EPA 7: Clinical questions to advance patient care	Post e-visit reflection Virtual journal clubs Student-led inquiry projects
EPA 8: Give or receive a patient hand-off	Virtual standardized patients and Objective Structured Clinical Examinations [8,9]
EPA 9: Collaborates as a member of an interprofessional team	Interdisciplinary rounds Teleconsults [4] Tumor board [17] Group discussions with other health-professional schools
EPA 10: Recognize urgent or emergent situations	Clinical e-visit Telestroke team [18] Teletrauma team [19] COVID-19 call centers, forward triage response team [4]
EPA 11: Obtain informed consent for tests and/or procedures	Clinical e-visits in surgery, surgical subspecialties, obstetrics and gynecology, etc [20]
EPA 12: General procedures of a physician	Online procedure courses, augmented and virtual reality simulations, including CPR^b^ training and ultrasound techniques [20] Participation in live-streamed surgical theaters [20]
EPA 13: Identify system failures and contribute to culture of safety and improvement	Post e-visit reflection Quality improvement training Student reflections

^a^e-visit: electronic visit.

^b^CPR: cardiopulmonary resuscitation.

By integrating multiple EPAs, a single e-visit could provide students exposure to skills that they will employ throughout their careers. A telemedicine student visit could be similar to most other ambulatory student clinic visits. To ease patient burden and reduce confusion, ideally the patient, the medical student, and the physician would all be present concurrently at the e-visit. The attending could observe the student lead as much of the visit as possible, with a seamless transition when the attending takes lead of the conversation. Using telehealth technology, students could gather a history and perform a virtual physical exam (EPA 1), focusing on communication skills, rapport building, and functional physical exam maneuvers. Students could summarize their findings (EPA 6) to both the patient and attending in patient-friendly language. Depending on the comfort level of the patient, the physician could lead the student through a discussion of the possible diagnoses (EPA 2), potential diagnostic tests (EPA 3), or orders (EPA 4). This style would mimic “family-centered rounds,” providing the student the opportunity to think through an assessment and plan with direct patient input. Alternatively, the physician and student could discuss the clinical reasoning and key academic concepts after the patient’s call. Following the visit, the medical student would then document the encounter (EPA 5). To augment these visits, medical students could research topics inspired by patient cases (EPA 7) and discuss the impacts of telemedicine on health care (EPA 13). These examples show how the e-visit can provide in-depth, high-quality education. A single e-visit can provide the opportunity to teach medical students invaluable skills that they would otherwise not be able to obtain from reading case studies.

With the expansion of novel telemedicine applications into more fields, including procedural fields, students can continue to learn foundational knowledge through engagement in telemedicine even beyond the stereotypical telemedicine visit. In fact, involvement in virtual opportunities exist for all EPAs (Table 1), thus making telemedicine a powerful supplement to the traditional inpatient and outpatient clinical curriculum. In surgery and obstetrics and gynecology, students could assist with pre- and postoperative e-visits (EPA 11) [20]. Students can become educators for patients, by researching a topic such as diabetes nutrition strategies (EPA 7) and then leading a group virtual session with patients (EPA 6). Within the context of inpatient encounters, the aforementioned outpatient recommendations can be further adapted to the inpatient setting. For example, students could participate in virtual consults by talking to admitted patients via a tablet or other similar equipment following the above format (EPAs 1-6). In pathology and radiology, screen-sharing technologies could allow students to become involved in review of slides and imaging remotely (EPAs 2 and 3). Unique experiences such as opportunities to triage urgent cases may exist for hospitals that have telestroke [18] or teletrauma teams (EPA 10) [19,20], or to participate in virtual interdisciplinary rounds (EPA 9) [17]. Students could benefit from participating in as few as one opportunity to augment their online learning. Furthermore, with so many clinicians going online, the use of telemedicine in education is not dependent on a single department. With telemedicine opportunities now available in almost every field, students have the opportunity to learn a variety of skills in multiple specialties.

Even if students are to return to the clinical setting soon, traditional clerkships may look different for them. For example, some students may have continued needs for self-quarantining while others may return to the hospital. For students in hospitals, there will likely still be patients admitted for COVID-19. In this context, telemedicine may provide a way to resume medical student education while maintaining a safe environment. For example, students can participate in consults or rounds without stepping into patient rooms through the use of tablets [4,5]. In fact, this is already a strategy for our institution’s burn center during multidisciplinary rounds. As previously stated, even as clerkships return and social distancing measures are relaxed, the high use of telemedicine will likely persist. Therefore, students could continue to participate in these e-visits during their clerkships. The vast array of telemedicine initiatives can be utilized to augment traditional clerkships, providing students with broader access to diverse learning opportunities.

## Concluding Thoughts

As telemedicine is further implemented, clinical skills unique to remote care have become necessary. The rapid adoption of telemedicine due to the COVID-19 outbreak can serve as an opportunity to augment medical education curricula and to continue to provide medical students with critical educational opportunities when in-person encounters are limited. If hospital systems are already building and expanding videoconferencing and e-visit tools for providers, why not include students as well?

## Abbreviations

AAMC: Association of American Medical Colleges
AMA: American Medical Association
COVID-19: coronavirus disease
CPR: cardiopulmonary resuscitation
EHR: electronic health record
e-prescribing: electronic prescribing
EPA: Entrustable Professional Activity
e-visit: electronic visit
HIPAA: Health Insurance Portability and Accountability Act
